# Impact of health system challenges on prostate cancer control: health care experiences in Nigeria

**DOI:** 10.1186/1750-9378-6-S2-S5

**Published:** 2011-09-23

**Authors:** Olufemi J Ogunbiyi

**Affiliations:** 1 Department of pathology, University College Hospital/ College of medicine, University of Ibadan, P.M.B. 5116, Ibadan, Oyo state, Nigeria

## Abstract

Prostate cancer is the second most frequently diagnosed cancer of men (913 000 new cases, 13.8% of the total) and the fifth most common cancer overall. Prostate cancer is the sixth leading cause of death from cancer in men (6.1% of the total).

The incidence of prostate cancer in Nigerian men is believed to be on the increase and it had become the number one cancer in 1999, constituting 11% of all male cancers in the population served by the Ibadan cancer Registry. Studies from Ibadan and from other sites in Nigeria (Benin, Calabar, Kano, Lagos, Maiduguri, and Zaria) have shown an increasing incidence of prostate cancer accounting for anything between 6 and 12 % of total cancers in these centres and up to about 18% of prostatic neoplasms in some. Most patients present in late stage disease, and the mortality is high.

It is uncertain whether there is a biophysical component to the increased incidence of prostate cancer in Nigerian and other West African men although a few studies point in this direction.

It appears there is inadequate information regarding the incidence and mortality of prostate cancer, and that health care professionals do not routinely provide information regarding the importance of screening for prostate cancer before age 50 for high-risk populations.

The Revised National health Policy for Nigeria (Sept 2004) has as its long term goal ‘to provide the entire population with adequate access, not only to primary health care but also to secondary and tertiary services through a well functioning referral system’ and also “Ensuring equitable distribution of human resources for healthcare delivery between urban and rural areas, including difficult terrain, such as mountainous, riverine and inaccessible areas of the country.”

At the moment however, Public expenditure on health is less than $8 per capita, compared to the $34 recommended internationally. Private expenditures are estimated to be over 70% of total health expenditure, most of this from out-of-pocket. Yet there is endemic poverty. The National Health Insurance scheme (NHIS) is presently severely limited in its allowances and certainly so for cancer care.

**Conclusions:**

The following recommendations are therefore made:

* Establishment of community outreaches for education and screening.

* Improved completeness of records to understand the real burden of disease and funding studies to explore biophysical components that may be important in racial differences for this disease.

* Increased access by increasing numbers of specialists required for clinical assessment and management.

* Increase laboratory diagnostic support

* Improved availability of drugs for the treatment of prostate cancer cases.

## Background

Prostate cancer is the second most frequently diagnosed cancer of men (913 000 new cases, 13.8% of the total) and the fifth most common cancer overall. With an estimated 258 000 deaths in 2008, prostate cancer is the sixth leading cause of death from cancer in men (6.1% of the total). [1]

Incidence rates for prostate cancer vary by more than 25-fold worldwide, with the highest rates seen in Australia/New Zealand (104.2 per 100,000), Western and Northern Europe, and Northern America. This is probably because prostate specific antigen (PSA) testing and subsequent biopsy has become widespread in those regions. PSA screening has been recommended for Caucasian, Asian, and Hispanic men at age 50 but because one of the most important risk factors for prostate cancer in the United States is African-American descent, African-Americans are screened from age 45. [2] There is less variation in mortality rates worldwide (10-fold) than is observed for incidence, because PSA testing has a much greater effect on incidence than on mortality, and the number of deaths from prostate cancer is almost the same in developed and developing regions. [3] Mortality rates are generally high in predominantly black populations (Caribbean, 26.3 per 100,000 and sub-Saharan Africa, ASRs 18-19 per 100,000), very low in Asia (ASR 2.5 per 100,000 in Eastern Asia for example) and intermediate in Europe and Oceania.

## Methods

A review of available reports on the incidence and mortality of prostate cancer in Nigeria and West Africa, including modalities available for diagnosis and treatment.

## Results

The incidence of prostate cancer in Nigerian men is believed to be on the increase and it had become the number one cancer in 1999, constituting 11% of all male cancers, moving ahead of hepatocellular carcinoma in incidence and mortality in the population served by the Ibadan cancer Registry. [4]

Studies from Ibadan and from other sites in Nigeria (Benin, Calabar, Kano, Lagos, Maiduguri, and Zaria) have shown an increasing incidence of prostate cancer. In one study, there was a 7.7-fold increase over a 10-year period. Prostate cancer accounts for anything between 6 and 12 % of total cancers in these centres and up to about 18% of prostatic neoplasms in some. Interestingly the average age from the different subpopulations is in the 7^th^ decade and the ages range from 40+ to 90+. [5-10]

**Figure 1:**
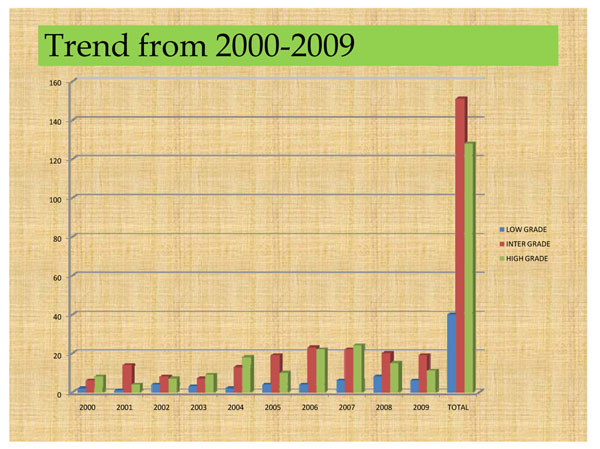
Trend in incidence from 2000- 2009(Ibadan cases only)

In all sites, patients present in late stages of disease with an average duration of symptoms of 6-8 months, and tend to die within 2 – 3 years, except for a few exceptions (one patient was alive 46 months after presenting with what was diagnosed as orbital metastasis of prostate cancer). [11] There however seems to be a gradual shift from predominantly high grade tumours towards intermediate grade tumours in the data from Ibadan (Figure 1). Incidence data is however still inadequate.

Because of the usually late presentation, most reported patients have been treated with orchidectomy or other forms of hormonal intervention by drugs. It is known that they eventually become resistant to these hormonal manipulations. For metastatic disease, radiation therapy has been used. [12] Again, reports of survival studies are nearly unavailable.

## Discussion

This disparity in outcome, when compared to African Americans, raises the following issues about prostate cancer in Nigeria and West Africa in general:

*Is there adequate information regarding the incidence and mortality of prostate cancer? The latest cancer incidence publication of the International Agency for research on Cancer (IARC) shows no representation of cancer data from West Africa. There are not many cancer registries that submit reliable data that meet the inclusion criteria for that publication and figures from GLOBOCAN are mainly extrapolative. There are only two population based cancer registries in Nigeria and data from these have not been accepted recently by the CIV (Cancer Incidence in Five Continents) group of the IARC. [13]

*Do health care professionals routinely provide information regarding the importance of screening for prostate cancer before age 50 for high-risk populations? As far as we know, there is no established prostate cancer screening programme in Nigeria and many of the West African Countries. There is a recent attempt as establishing a programme in Senegal. The challenges are that there are not enough trained Urologists in any of these countries, most of these are located in practices within the big urban centres, and there is inadequate support for follow up treatment for those that might be detected.

*Is there a biophysical component to the increased incidence of prostate cancer in Nigerian and other West African men? There is some literature in support of this at the moment but the extent of this needs further enquiry. Ukoli et al found central adiposity to be a positive predictor of elevated PSA amongst a subset of Nigerians in one study, after adjusting for age and enlarged prostate. [14] In addition, molecular mechanisms have been shown involved in racial disparities in prostate cancer. [15]

*Can primary prevention interventions, such as eating less fat and increasing fruit and vegetable consumption, reduce the incidence of prostate cancer in these populations?

*Do current screening techniques of prostate specific antigen (PSA) testing and digital rectal examination (DRE) adequately detect early prostate cancer?

There is a whole controversy as to the exact usefulness of screening techniques but there is agreement that a combination of PSA measurement, digital rectal examination (DRE), and prostatic ultrasound scanning will significantly improve detection rates for early cancer.

**Figure 2:**
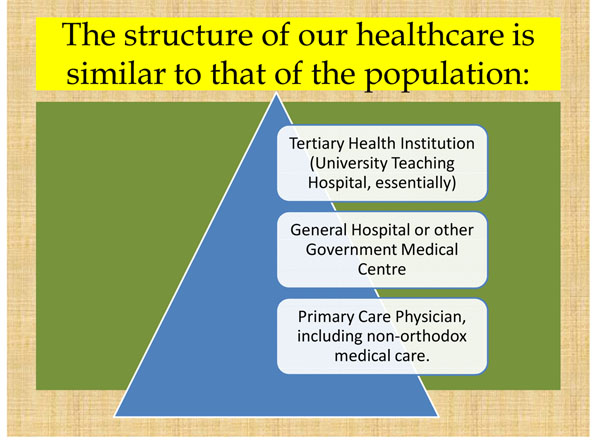
The distribution of healthcare facilities

With the pyramidal structure of our healthcare system (Figure 2)in which only the smallest proportion of the population access tertiary care in urban cities, whereas the larger proportion live in the villages, there is obviously a disproportionate access to specialist care and laboratory support for the much needed screening programmes.

There is a Revised National health Policy for Nigeria (Sept 2004) that is derived from the policy promulgated in 1988 which seems to acknowledge these challenges because ‘Its long term goal is to provide the entire population with adequate access, not only to primary health care but also to secondary and tertiary services through a well functioning referral system.’ On p.31 of the National Health policy document, under ‘national health human resource development 2.’ It states “Ensuring equitable distribution of human resources for healthcare delivery between urban and rural areas, including difficult terrain, such as mountainous, riverine, and inaccessible areas of the country.” [16]

At the moment however, Public expenditure on health is less than $8 per capita, compared to the $34 recommended internationally. Private expenditures are estimated to be over 70% of total health expenditure, most of this from out-of-pocket. Yet there is endemic poverty. There is a National Health Insurance scheme (NHIS) presently, which is severely limited in its allowances and certainly so for cancer care.

## Conclusions

The following recommendations seem appropriate at this time and are therefore made:

* Establishment of community outreaches for education and screening.

* Improved completeness of records to understand the real burden of disease

* Increased access to care by increasing the numbers of specialists required for clinical assessment and management.

* Increase laboratory diagnostic support

* Improved availability of drugs for the treatment of cancers.

## Competing interests

The Author has no financial or non-financial competing interests in relation to the contents of this manuscript.
